# Repetitive Hyperbaric Oxygenation Attenuates Reactive Astrogliosis and Suppresses Expression of Inflammatory Mediators in the Rat Model of Brain Injury

**DOI:** 10.1155/2015/498405

**Published:** 2015-04-20

**Authors:** Irena Lavrnja, Ana Parabucki, Predrag Brkic, Tomislav Jovanovic, Sanja Dacic, Danijela Savic, Igor Pantic, Mirjana Stojiljkovic, Sanja Pekovic

**Affiliations:** ^1^Department of Neurobiology, Institute for Biological Research “Sinisa Stankovic”, University of Belgrade, 11060 Belgrade, Serbia; ^2^Institute of Medical Physiology “Richard Burian”, School of Medicine, University of Belgrade, 11000 Belgrade, Serbia; ^3^Centre for Hyperbaric Medicine, 11040 Belgrade, Serbia; ^4^Institute of Physiology and Biochemistry, Faculty of Biology, University of Belgrade, 11001 Belgrade, Serbia

## Abstract

The exact mechanisms by which treatment with hyperbaric oxygen (HBOT) exerts its beneficial effects on recovery after brain injury are still unrevealed. Therefore, in this study we investigated the influence of repetitive HBOT on the reactive astrogliosis and expression of mediators of inflammation after cortical stab injury (CSI). CSI was performed on male Wistar rats, divided into control, sham, and lesioned groups with appropriate HBO. The HBOT protocol was as follows: 10 minutes of slow compression, 2.5 atmospheres absolute (ATA) for 60 minutes, and 10 minutes of slow decompression, once a day for 10 consecutive days. Data obtained using real-time polymerase chain reaction, Western blot, and immunohistochemical and immunofluorescence analyses revealed that repetitive HBOT applied after the CSI attenuates reactive astrogliosis and glial scarring, and reduces expression of GFAP (glial fibrillary acidic protein), vimentin, and ICAM-1 (intercellular adhesion molecule-1) both at gene and tissue levels. In addition, HBOT prevents expression of CD40 and its ligand CD40L on microglia, neutrophils, cortical neurons, and reactive astrocytes. Accordingly, repetitive HBOT, by prevention of glial scarring and limiting of expression of inflammatory mediators, supports formation of more permissive environment for repair and regeneration.

## 1. Introduction

Increased inflammatory reaction is elicited after traumatic brain injury (TBI) in areas proximal and distal to the locus of primary insult [1–3]. An invasion of macrophages and neutrophils into the impact area is triggered, and they initiate much of the inflammation and swelling in damaged areas that can directly affect the outcome after TBI [4, 5]. It is well documented that the basic mechanisms underlying neuroinflammatory cascade involves activation and ligation of CD40 ligand (CD40L, also termed CD154, or GP39) and its counter receptor CD40 [6]. On the surface of vascular endothelial cells, CD40/CD40L ligation upregulates production of ICAM-1 (CD54), an adhesion molecule that is important for transendothelial migration of neutrophils and propagation of inflammation [7, 8]. Considering that CD40/CD40L dyad fosters neuroinflammation, some studies suggest that CD40/CD40L interaction may be involved in modulating the outcome from injuries of the brain [9–11]. Strategies aimed at suppressing CD40/CD40L/ICAM-1 expression, therefore, may attenuate inflammation and neuronal damage after TBI, which will ultimately be of benefit in recovery [12].

Another obstacle for a successful recovery after TBI is the existence of nonpermissive glial scar, which prevents axonal sprouting and the establishment of new neural circuits but also isolates intact central nervous system (CNS) tissue from secondary lesions [13, 14]. The glial scar consists mainly of reactive astrocytes, which upon TBI undergo reactive astrogliosis involving cell proliferation, hypertrophy, and an enhancement of immune-modulating capacities [9, 15]. Therefore, downregulation of reactive astrogliosis will produce permissive environment for neurite outgrowth and formation of new synaptic connections and decrease inflammation.

Posttraumatic imbalance between cerebral oxygen delivery and cerebral oxygen consumption is also one of the consequences of TBI [16]. Among different therapies, several animal and clinical studies have demonstrated promising effects of hyperbaric oxygenation (HBO) after various types of brain injuries [17–19]. Over the years treatment with hyperbaric oxygen (HBOT) has become the primary therapy for a variety of clinical conditions [20], including dose-dependent effects on inflammation, angiogenesis, blood-brain barrier (BBB) integrity, and wound healing [17, 21–23]. In our recently published papers, we have demonstrated that repetitive application of HBOT after cortical injury prevented neurodegeneration due to the reduction of oxidative stress [24] and improved neuroplastic responses, which contributed to the recovery of motor performances and sensorimotor integration in rats [25].

Although hyperbaric medicine is advancing, and understanding of its beneficial effects in many diseases has been improved, the studies exploring possible anti-inflammatory effects of HBO after stab brain injury are lacking. Therefore, in this study we explored the potential of HBOT to reduce astrocyte-mediated inflammatory response to brain injury. Our results indicate that repetitive HBOT limits production of inflammatory mediators (CD40, CD40L, and ICAM-1), prevents astrocyte activation, and reduces glial scar formation.

## 2. Materials and Methods

### 2.1. Animals

The experiments were performed on adult male Wistar rats (10 weeks old). All experimental procedures received prior approval from the Ethical Committee of the School of Medicine, University of Belgrade (number 3027/2), and were in compliance with the Directive 2010/63/EU on the protection of animals used for experimental and other scientific purposes. At each stage of the experiment, all possible steps were taken to minimize animal suffering and to reduce the number of animals used. At the beginning of the experiments, there was no statistically significant difference in animal's body weight (250 ± 30 g), within the group as well as between the groups. All animals were housed in fours per cage (55 × 35 × 30 cm), under standard conditions (23 ± 2°C, 50–60% relative humidity, 12 h/12 h light/dark cycle with lights on at 07:00, with free access to food and water). The period of adaptation lasted for one week. After the acclimatization period, a cohort of 72 animals was divided into 6 experimental groups in a randomized fashion (*n* = 12 per group): control group (C), intact rats; control HBO group (CHBO), intact rats subjected to the HBO protocol for 10 consecutive days; sham group (S), the animals that underwent surgical procedure without skull opening and were sacrificed 10 days after injury (dpi); sham HBO group (SHBO), the animals that underwent sham surgery and were subjected to the HBO protocol for 10 consecutive days; lesion group (L), the animals that passed cortical stab injury (CSI) and were sacrificed at 10th dpi; and lesion HBO group (LHBO), CSI rats subjected to the HBO protocol for 10 consecutive days.

### 2.2. Cortical Stab Injury (CSI)

Before starting the surgical procedure, rats were weighted and anesthetized with ether. Preoperatively, after the onset of anesthesia, the rat's scalps were shaved and then they were placed in stereotaxic frame. The head was incised using a sterile scalpel blade and the bleeding was minimized using cotton swabs. Using a hand held 1 mm wide dental drill the skull was surgically exposed, and stab injury to the left cortex was performed at the following coordinates: 2 mm posterior to the bregma and 2 mm lateral from the midline and to a depth of 2 mm into the brain [26]. The incision was closed with sutures. All rats survived the procedure of induction of brain injury. Rats from the sham control groups underwent anesthesia and the same surgical procedure except inflicting stab injury. Intact animals were untreated age-matched controls. After the surgery, the rats were kept at warm and left up to 2 hours to recover before they were included in the hyperbaric oxygen protocol. All animals survived surgical procedure and quickly recovered after operation.

### 2.3. HBO Treatment (HBOT) Protocol

As described previously [25] the rats were placed into experimental HBO chambers and exposed to 100% oxygen according to the following protocol: 10 minutes of slow compression, 2.5 atmospheres absolute (ATA) for 60 minutes, and 10 minutes of slow decompression, once a day during 10-day period. Upon reaching the desired pressure, the flow of oxygen was reduced to maintain constant pressure while allowing the flow out of the chamber. This constant exchange accompanied by a tray of calcium carbonate crystals was used to reduce the accumulation of CO in the chamber environment. This protocol corresponds to a standard hyperbaric oxygen treatment that is routinely used in the clinical setting of Center for Hyperbaric Medicine, Belgrade, Serbia [25, 27], and is in a line with recommendations of The Committee of the Undersea and Hyperbaric Medical Society that a treatment pressure only from 2.4 to 3.0 ATA should be used as appropriate [28], Each exposure was started at the same hour to exclude any confounding issues associated with the changes in biological rhythm. Body temperature was not changed significantly after the HBOT.

### 2.4. Tissue Processing for Gene Expression Analyses

Four rats of each group were taken for gene expression analyses. After the end of treatment protocol (at 10th day after surgery) animals were sacrificed by decapitation under deep ether anesthesia. Immediately after decapitation, the brains were quickly removed from the skull. As described in our recently published paper [24], from injured (left) cortices 2 mm sections around the center of lesion were dissected on ice, rapidly frozen in liquid nitrogen, and stored at −80°C until further processing. The same piece of tissue was dissected from the left cortices of sham and intact controls. Total cellular RNA was isolated from the brain tissue using TRIzol isolation method (Invitrogen Life Technologies, Carlsbad, CA, USA) as per manufacturer's direction. RNA purity and concentration were insured by gel visualization and spectrophotometrically by calculating the ration between the absorbance at 260 nm and 280 nm. The absorbance ratio for all samples ranged between 1.8 and 2.1. RNA was transcribed to cDNA using High Capacity cDNA Reverse Transcription Kit (Applied Biosystems) according to manufacture instructions.

### 2.5. Real Time (RT)-PCR

TaqMan PCR reactions were performed with Assay-on-Demand Gene Expression Products (Applied Biosystems) for GFAP (Assay ID Rn00566603_m1) and ICAM-1 (Assay ID Rn 00564227). PCR reactions were carried out in the ABI Prism 7000 Sequence Detection System at 50°C for 2 min, at 95°C for 10 min, followed by 40 cycles, at 95°C for 15 s, and at 60°C for 1 min. A reference, endogenous control, was included in every analysis to correct the differences in interassay amplification efficiency, and the expression of each gene was normalized to GAPDH (glyceraldehyde-3-phosphate dehydrogenase) expression. The obtained results were analyzed by RQ Study Add ON software for 7000 v 1.1 SDS instrument (ABI Prism Sequence Detection System) with a confidence level of 95% (*P* < 0.05). The cDNA products were used for quantitative real-time PCR carried out with the SYBR Green PCR Master Mix gene expression assay (Applied Biosystems, UK) according to the manufacturer's instructions. Reactions were conducted in the ABI Prism 7000 Sequence Detection System (Applied Biosystems, USA). Relative gene expression was calculated by comparing CT value of the gene of interest to the CT value of GAPDH, internal control (the 2-ΔΔCT method). The following primer pairs from Invitrogen, Germany, were used: GAPDH (157 bp product): forward, TGGACCTCATGGCCTACAT; reverse GGATGGAATTGTGAGGGAGA; and vimentin (87 bp product): forward CGTACGTCAGCAATATGAAAGTGTG; reverse TCAGAGAGGTCAGCAAACTTGGA. GAPDH and vimentin were amplified at an annealing temperature of 60°C for 40 cycles. The PCR products were run on 2% agarose gels and visualized under UV light (data not shown).

### 2.6. Western Blot Analysis

After decapitation brains were removed, left cortices were dissected and pooled from four animals. The selected tissue was homogenized with a hand-held pestle in sodium dodecyl sulfate (SDS) sample buffer (10 mL/mg tissue), which contained a cocktail of proteinase and phosphatase inhibitors. The electrophoresis samples were heated at 100°C for 5 min and loaded onto 10% SDS-polyacrylamide gels with standard Laemmli solutions (Bio-Rad Laboratories, CA, USA). The proteins were blotted onto a polyvinylidene difluoride membrane (PVDF, Immobilon-P, Millipore, Billerica, MA, USA). The membranes were placed in a blocking solution, which contained Tris-buffered saline with 0.02% Tween (TBS-T) and 5% nonfat dry milk, for 1 h, and incubated overnight under gentle agitation with primary antibody rabbit anti-GFAP (1 : 7000 Dako, Glostrup, Denmark), mouse antivimentin (1 : 5000, Dako, Glostrup, Denmark), goat anti-CD40L (1 : 1000; Santa Cruz Biotechnology, Santa Cruz, CA, USA), and mouse anti-*β*-actin (1 : 1000; Sigma, St. Louis, MO, USA), respectively. Bound primary antibodies were detected with a horseradish peroxidase- (HRP-) conjugated anti-rabbit, anti-mouse, or anti-goat secondary antibody (1 : 5000; Santa Cruz Biotechnology, Santa Cruz, CA, USA). Between each step, the immunoblots were rinsed with TBS-T. Immunoreactive bands were visualized on X-ray films (Kodak) using chemiluminescence. Optical densities of immunoreactive bands from 4 independent blots were calculated in Image Quant program. The densities of GFAP, vimentin, CD40L, and *β*-actin immunoreactive bands were quantified with background subtraction. Squares of identical sizes were drawn around each band to measure density, and background near that band was subtracted. For each blot, optical densities were normalized against *β*-actin levels.

### 2.7. Tissue Processing for Immunohistochemical and Immunofluorescence Analyses

For immunohistochemical and immunofluorescence analyses, brains (four per group) were quickly removed and fixed in 4% paraformaldehyde in 0.1 M phosphate buffer (PBS), pH 7.4 for 12 hours at 4°C. For cryoprotection brain tissue was transferred into the graded sucrose (10–30% in 0.1 M PBS, pH 7.4). The brains were frozen in 2-methylbutane and kept at −80°C until sectioning on cryotome. Coronal sections (25 *μ*m thick) of the left cortex were collected serially, mounted on superfrost glass slides, dried for 2 h at room temperature, and stored at −20°C until staining. Immunohistochemical and immunofluorescence data were determined on 5–7 slides per rat. No significant differences in immunoreactivity between C, CHBO, S, and SHBO controls were observed (data are shown as supplemented material in Supplementary Material available online at http://dx.doi.org/10.1155/2015/498405).

### 2.8. Immunohistochemistry

Single labeling for GFAP, vimentin, CD40, CD40L, and ICAM-1 was performed according to the standard procedure. Briefly, after neutralization of endogenous peroxidase with 0.3% H_2_O_2_ in methanol for 20 min at room temperature and washing in PBS, the sections were blocked by 5% normal donkey serum (Sigma, Germany) in order to reduce non-specific binding. Sections were incubated overnight at 4°C with primary antibodies: rabbit anti-GFAP (1 : 500; Dako, Glostrup, Denmark); mouse anti-vimentin (1 : 500; Dako, Glostrup, Denmark); rabbit anti-CD40, goat anti-CD40L; and goat anti-ICAM1 (1 : 100; Santa Cruz Biotechnology, Santa Cruz, CA, USA). Using appropriate peroxidase linked secondary antibody (1 : 200, Santa Cruz Biotechnology, Santa Cruz, CA, USA), the products of immunoreactions were visualized with 3′3-diaminobenzidine (DAB, Dako, Glostrup, Denmark) according to manufacturer's instructions. The specificity of the staining was tested on the sections in the second dish by omission of the primary specific antibodies. No immunoreactive products were found on these sections. After dehydration and clearing, sections were mounted with DPX Mounting medium (Fluka) and examined under Carl Zeiss Axiovert microscope (Zeiss, Gottingen, Germany). In order to quantify the reduction of glial scar size after HBOT we measured GFAP/vimentin DAB staining intensity as described in [29]. Images were captured under the same conditions on a Zeiss Axiovert (Zeiss, Jena, Germany). Then, image colors were inverted in Photoshop 7.0 (Adobe Systems, San Jose, CA), and luminosity of glial scar (histogram function in Photoshop) was determined as mean intensity of five areas of a fixed size (500 × 200 *μ*m) around the lesion site. The data were collected from 3 consecutive brain slices per rat and four rats per group.

### 2.9. Immunofluorescence Analysis

Colocalization of ICAM-1, CD40 and CD40L with specific markers of neuronal cell bodies (neuronal specific nuclear protein, NeuN), dendrites (Microtubule-associated protein 2, MAP-2),reactive astrocytes (GFAP), microglia (ionized calcium binding adapter molecule 1, Iba1), and neutrophils (R-MC46) was examined with double immunofluorescence labeling. Cells nuclei were stained with DAPI (diamidino-2-phenylindole). Antigen retrieval step in the heated citrate buffer (pH 6) and tissue permeabilization with 0.3% Triton X-100 in PBS were performed in order to enhance the staining, where it was necessary. Normal donkey serum (Sigma) was used for blocking of unspecific labeling as 5% solution in PBS. For immunofluorescent double-labeling, section was incubated first with goat anti-ICAM-1 (1 : 100; Santa Cruz Biotechnology, Santa Cruz, CA, USA), goat anti-CD40L (1 : 100; Santa Cruz Biotechnology, Santa Cruz, CA, USA), or rabbit anti-CD40 (1 : 100; Santa Cruz Biotechnology, Santa Cruz, CA, USA), followed by specific cell marker antibodies: rabbit anti-GFAP (1 : 500; Dako, Glostrup, Denmark), mouse anti-NeuN (1 : 200, Millipore, USA), mouse anti-MAP-2 (1 : 100; Sigma, Germany), goat anti-Iba1 (1 : 400, Abcam, Cambridge, MA, USA), or mouse anti-R-MC46 (1: 200, a kind gift of prof. Sasa Vasilijic, Institute for Medical Research, Military Medical Academy, Belgrade, Serbia). Mouse anti-R-MC46 was used to identify neutrophils [30]. Immune complexes were visualized with donkey anti-goat Alexa Fluor 488 and donkey anti-rabbit IgG Alexa Fluor 555 (1 : 250, Invitrogen, Carlsbad, CA, USA) or with donkey anti-rabbit IgG Alexa Fluor 488 and donkey anti-mouse Alexa Fluor 555 (1 : 250, Invitrogen, Carlsbad, CA, USA), while nuclei were counterstained with DAPI (Invitrogen, Grand Island, NY, USA). Control sections were also incubated with appropriated secondary antibodies without the primary antibody as a negative control. The sections were mounted in mowiol (Calbiochem, San Diego, CA) and examined under the Carl Zeiss Axiovert fluorescent microscope (Zeiss, Gottingen, Germany) equipped with camera and EC Plan-Apochromat 100x objective, using the ApoTome software module for generating optical sections through fluorescence samples. Images were acquired on magnifications as indicated.

### 2.10. Statistical Analysis

All data were collected and analyzed by researcher blinded to the surgery and HBOT protocol used. Results were presented as mean ± SEM. Significance of difference between the data obtained for different groups was determined using Student's *t*-test. The values of *P* ≤ 0.05 were considered statistically significant.

## 3. Results

It is important to note that there was no statistically significant difference between data obtained for C, CHBO, S, and SHBO groups and, therefore, for immunohistochemical analysis all comparisons were done with respect to intact controls (data are shown as supplemented material).

### 3.1. The Response of Astroglial Cells to HBOT following CSI

To investigate the effect of repetitive HBOT on reactive astrogliosis and glial scar formation after CSI we followed expression of GFAP and vimentin (as markers of astrocytic activation) at gene, protein, and tissue levels in the left cortices.

#### 3.1.1. HBOT Reduces Gene, Protein, and Tissue Expression of GFAP after CSI

**Figure 1:**
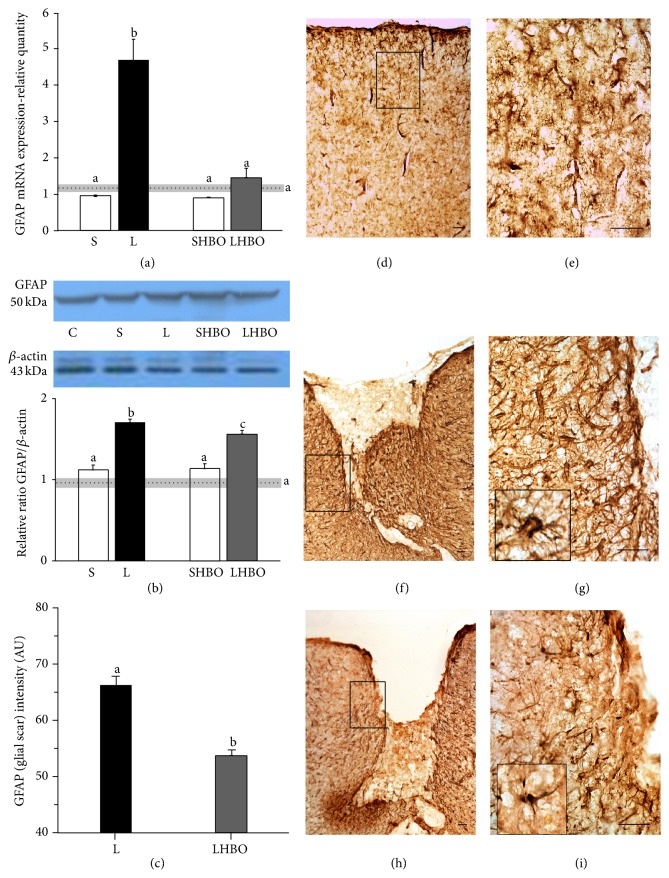
Effect of repetitive HBOT on GFAP expression after CSI. CSI provoked upregulation of GFAP expression both at mRNA (a) and protein (b) levels in the injured cortex compared to intact control and sham-operated animals, while repetitive HBOT reduced levels of GFAP mRNA to those detected in control groups. (a) Bars represent mean ± SEM of GFAP mRNA (relative to GAPDH). (b) Immunoblot analysis showed that GFAP was present as a single band with a molecular mass of about 50 kDa. Bars represent mean ± SEM of GFAP protein content (relative to *β*-actin). Samples are from 4 animals per each group. Dot line represents mean of GFAP mRNA or protein level ± SEM (gray area) measured in control animals. Letters indicate significance levels (*P* < 0.005) between lesioned (L) and intact control group, L versus sham control (S) group, and L compared to lesioned group subjected to the HBO protocol (LHBO). The groups not sharing a common letter are statistically different. Level of significance was analyzed using Student's *t*-test. (c) The luminosity of glial scar is obtained by measuring GFAP staining intensity around the lesion site and presented on histogram. ((d) and (e)) Throughout the cortex of intact rats a small number of fibrous GFAP^+^ astrocytes is seen. ((f) and (g)) At 10 days after injury a huge number of reactive astrocytes with pronounced hypertrophy of cell body and processes ((g) inset) form glial scar around the lesion site. ((h) and (i)) Ten successive HBOT significantly reduced glial scar formation, and the majority of astrocytes attained fibrous morphology ((i) inset). Rectangles indicate where the high magnification images are taken from. Scale bar = 50 *μ*m.

Real-time PCR analysis was used to evaluate the effect of HBOT and CSI on GFAP gene expression. After CSI, the mRNA level of GFAP was increased 4-fold (*P* < 0.005) in the injured cortex with respect to intact control and sham-operated animals. In contrast, repetitive HBOT induced downregulation of GFAP mRNA expression to levels detected in control groups (Figure 1(a)).

To assess the pattern of GFAP protein expression following CSI and HBOT, cortices were isolated 10 days after injury. Immunoblot analysis showed that GFAP was present as a single band with a molecular mass of about 50 kDa (Figure 1(b)). Statistically significant increase (*P* < 0.005) in GFAP expression was detected after CSI with respect to physiological control and sham-operated animals. However, repetitive HBOT induced a statistically significant (*P* < 0.05) decrease in GFAP expression.

Immunostaining with anti-GFAP antibody revealed a paucity of fibrous astrocytes with small cell bodies and long, thin processes throughout the cortex of intact rat (Figures 1(d) and 1(e)). CSI induced an increase of GFAP staining in reactive astrocytes 10 days after injury (Figures 1(f) and 1(g)). Noticed reactive astrocytes had pronounced hypertrophy of cell body and processes (Figure 1(g) inset), with extension of processes beyond the previous domains of individual astrocytes. Hence, there is overlapping of astrocytic processes without clear border between those processes. These astrocytes form glial scar along the boundaries to necrotic tissue (Figures 1(f) and 1(g)). After 10 successive HBOT glial scar formation was significantly reduced (Figures 1(h) and 1(i)). The majority of astrocytes had fibrous morphology (Figure 1(i) inset), similar to that observed in controls, and there was no overlapping of processes between astrocytes (Figures 1(h) and 1(i)). In order to quantify the reduction of glial scar size after HBOT we measured GFAP staining intensity around the lesion site. The luminosity of glial scar is presented on histogram (Figure 1(c)). HBOT induced statistically significant (20%, *P* < 0.005) reduction of glial scar.

#### 3.1.2. HBOT Downregulates Vimentin Gene, Protein, and Tissue Expression after CSI

**Figure 2:**
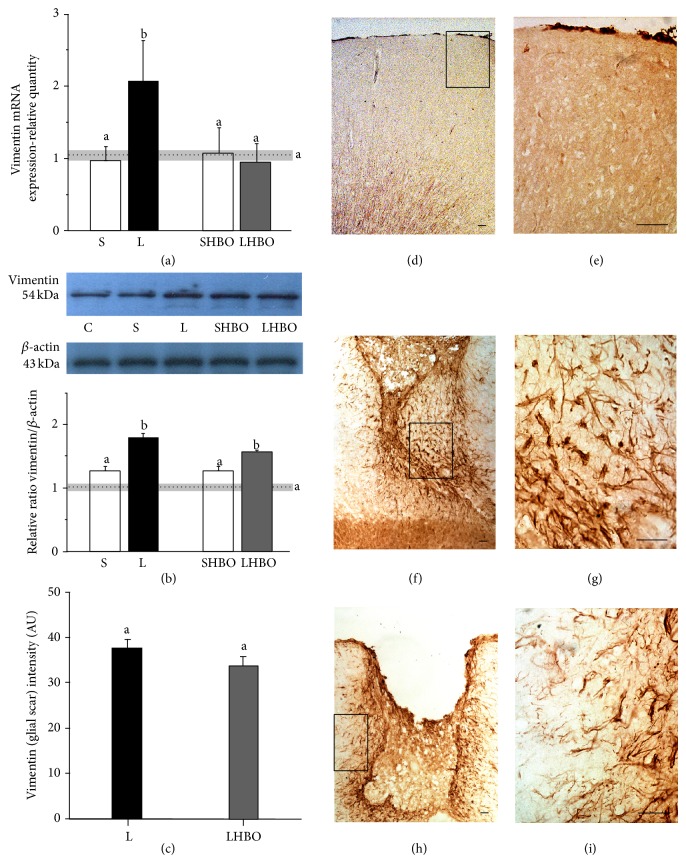
Repetitive HBOT reduces gene and tissue expression of vimentin after CSI. Upregulation of vimentin expression both at mRNA (a) and protein (b) levels is observed after CSI in the injured cortex compared to intact control and sham-operated animals. (a) Repetitive HBOT reduced levels of vimentin mRNA to those detected in control groups. Bars represent mean ± SEM of vimentin mRNA (relative to GAPDH). (b) Immunoblot analysis showed that vimentin was present as a single band with a molecular mass of about 57 kDa. HBOT slightly but not statistically significantly decreased vimentin expression. Bars represent mean ± SEM of vimentin protein content (relative to *β*-actin). Samples are from 4 animals per each group. Dot line represents mean of vimentin mRNA or protein level ± SEM (gray area) measured in control animals. Letters indicate significance levels (*P* < 0.005) between lesioned (L) and intact control groups, L versus sham control (S) group, and L compared to lesioned group subjected to the HBO protocol (LHBO). The groups not sharing a common letter are statistically different. Level of significance was analyzed using Student's *t*-test. (c) The luminosity of glial scar is obtained by measuring vimentin staining intensity around the lesion site and presented on histogram. ((d) and (e)) Vimentin immunoreactivity was negligible in the cortex of intact rats. ((f) and (g)) After CSI vimentin staining was significantly increased in the proximity to the lesion site. Vimentin^+^ astrocytes had enlarged cell bodies with thick processes. ((h) and (i)) After HBOT reactive astrocytes surrounded the lesion site as a narrow line. Rectangles indicate where the high magnification images are taken from. Scale bar = 50 *μ*m.

Similarly to GFAP, vimentin mRNA was also upregulated (2-fold, *P* < 0.005) in the injured cortices 10 days after the CSI compared to the control groups. After HBOT level of vimentin mRNA significantly (*P* < 0.005) dropped to the control values (Figure 2(a)).

Western blot analysis showed that vimentin was present as a single band with a molecular mass of about 57 kDa (Figure 2(b)). CSI induced statistically significant (*P* < 0.005) increase in vimentin expression compared to physiological control and sham-operated animals. However, after repetitive HBOT, a slight decrease in vimentin expression was observed, but it was not statistically significant.

Weak vimentin immunoreactivity was seen throughout the cortex of intact rat (Figures 2(d) and 2(e)). At the 10th day after CSI vimentin staining was significantly increased in close vicinity to the lesion site (Figures 1(f) and 1(g)) but less than GFAP. The cell bodies of vimentin^+^ astrocytes were enlarged with thickened processes, but without processes overlapping (Figure 2(g)). Repetitive HBOT reduces the number of reactive astrocytes to a narrow border around the lesion site (Figure 2(h)). Moreover, those astrocytes mostly had fibrous form (Figure 2(i)). However, quantification of glial scar composed of vimentin^+^ astrocytes did not show statistically significant reduction of glial scar after HBOT (Figure 1(c)).

### 3.2. HBOT Prevents ICAM-1 Expression after CSI at Gene and Tissue Levels

**Figure 3:**
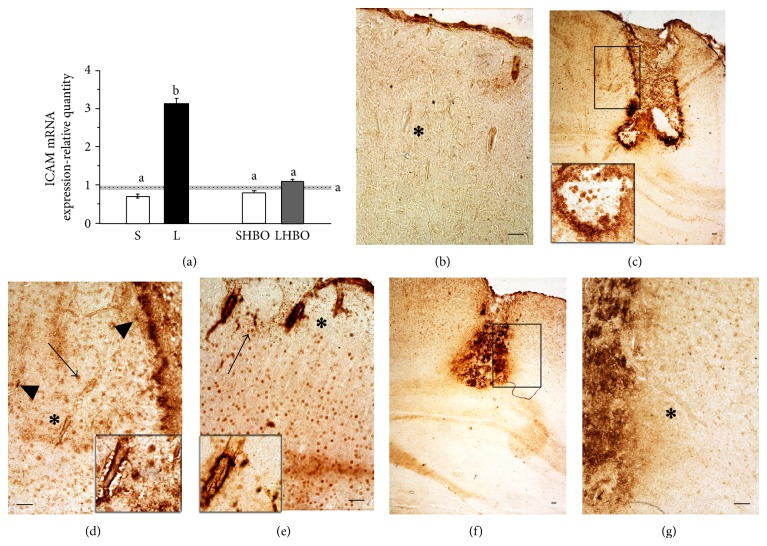
Repetitive HBOT reduces gene and tissue expression of ICAM-1 after CSI. (a) Repetitive HBOT attenuated injury-induced upregulation of ICAM-1 mRNA expression in the injured cortex. Bars represent mean ± SEM of ICAM-1 mRNA (relative to GAPDH). Samples are from 4 animals per each group. Dot line represents mean of ICAM-1 mRNA level ± SEM (gray area) measured in control animals. Letters indicate significance levels (*P* < 0.005) between lesioned (L) and intact control groups, L versus sham control (S) group, and L compared to lesioned group subjected to the HBO protocol (LHBO). The groups not sharing a common letter are statistically different. Level of significance was analyzed using Student's *t*-test. (b) In the control cortex ICAM-1 localization is present on the blood vessels (asterisk). ((c) and (d)) An increased ICAM-1 immunoreactivity is seen around the lesion site after the CSI. Heavy immunostaining of blood vessels is demonstrated both in ipsilateral (asterisk (d) and (e) inset) and contralateral cortex (asterisk (e) and (e) inset). The arrow denotes dark neuron-like cells in injured (d) and contralateral cortex (e). ICAM-1-positive neutrophils, microglia ((c), (c) inset), and astrocyte-like cells ((d), arrow head) were confined to the lesion area. (f) and (g) Repetitive HBOT reduced ICAM-1 immunoreactivity, while blood vessels were faintly stained ((g) asterisk). Rectangles indicate where the high magnification images are taken from. Scale bar = 50 *μ*m.

As shown in Figure 3(a) CSI induced 3.34-fold (*P* < 0.005) increase of ICAM mRNA levels in the injured cortex, compared to the control levels. Alternatively, 10 successive treatments with HBO significantly decreased (2.85-fold, *P* < 0.005) ICAM mRNA expression returning it to control levels (Figure 3(a)).

Throughout the control cortex weak ICAM-1 immunoreactivity was mostly associated with blood vessels (Figure 3(b)). After the CSI a robust ICAM-1 immunoreactivity occurred around the lesion site and in the nearby vasculature (Figures 3(c), 3(d), and 3(d) inset). Interestingly, heavy immunostaining of blood vessels was seen in the contralateral cortex as well (Figures 3(e) and 3(e) inset). In addition, dark neuron-like cells stained with anti-ICAM-1 were seen in both injured and contralateral cortexes (Figures 3(d) and 3(e)). In the proximity and within the lesion site a huge number of neutrophils and activated microglia, as well as astrocyte-like cells, were stained with ICAM-1 (Figures 3(c), 3(c), inset and 3(d)). On the other hand, after repetitive HBOT, ICAM1 immunoreactivity was diminished and localized only within the lesion site (Figures 3(f) and 3(g)). Blood vessels were faintly stained as in control cortical sections (Figure 3(g)).

**Figure 4:**
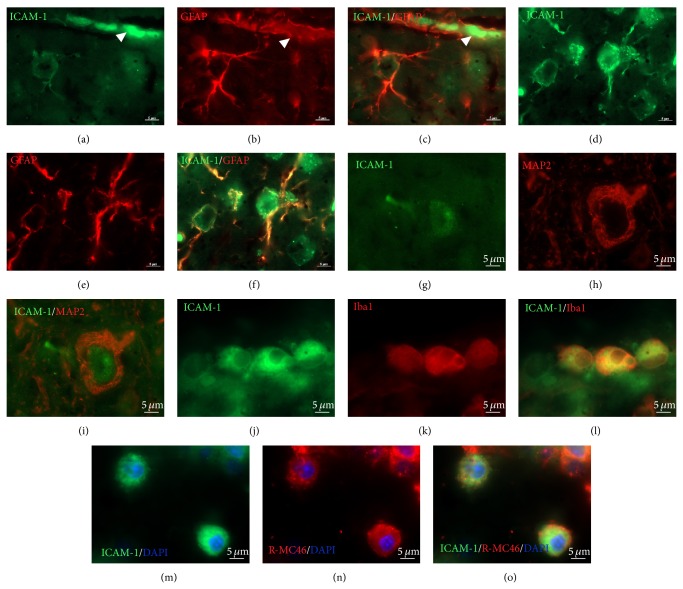
ICAM-1 (green fluorescence) colocalization with different cell types around and within the lesion site. ICAM-1 ((a) and (d)) and GFAP ((b) and (e) red fluorescence) coexpression was found in reactive astrocytes around the blood vessels ((c), arrow head) and in close vicinity to the lesion site (f). Colocalization of ICAM-1 (g) and MAP-2 ((h) red fluorescence) was detected mostly on neuronal cell bodies (i). ICAM-1 (j) colocalized only with activated microglia (Iba1, red fluorescence, (k)) clustered along the border to the lesion site and within the lesion site ((l) yellow fluorescence). Also, ICAM-1 completely overlaps with R-MC46^+^ neutrophils ((n) red fluorescence) only at the borders and within the lesion core ((o) yellow fluorescence)). Scale bar = 5 *μ*m.

To confirm colocalization of ICAM-1 immunoreactivity with different cell types double-immunofluorescence staining was performed. As it is shown at Figure 4 astrocytes that coexpress ICAM-1 (Figures 4(a) and 4(d), green) and GFAP (Figures 4(b) and 4(e), red) were found around the blood vessels (Figure 4(c)) and in close vicinity to the lesion site (Figure 4(f), yellow). Colocalization of ICAM-1 (Figure 4(g), green) and MAP-2 (Figure 4(h), red) was detected mostly on neuronal cell bodies (Figure 4(i)). Interestingly, only activated microglia clustered along the border to the lesion site and within the lesion site coexpress ICAM-1 (Figures 4(j)–4(l)). Similarly, only at the borders and within the lesion core strong ICAM-1 (Figure 4(m), green) immunofluorescence completely overlaps with R-MC46^+^ neutrophils (Figure 4(n), red) resulting in yellow fluorescence (Figure 4(o)).

### 3.3. Repetitive HBOT Significantly Prevented Neuroinflammation in the Injured Cortex

To assess the effect of HBOT on the spreading of neuroinflammation after CSI we performed immunostaining using anti-CD40 and anti-CD40L antibodies. Colocalization of CD40 and CD40L immunoreactivity with different cell types was visualized using double-immunofluorescence staining.

#### 3.3.1. HBOT Inhibits CD40 Expression after CSI

**Figure 5:**
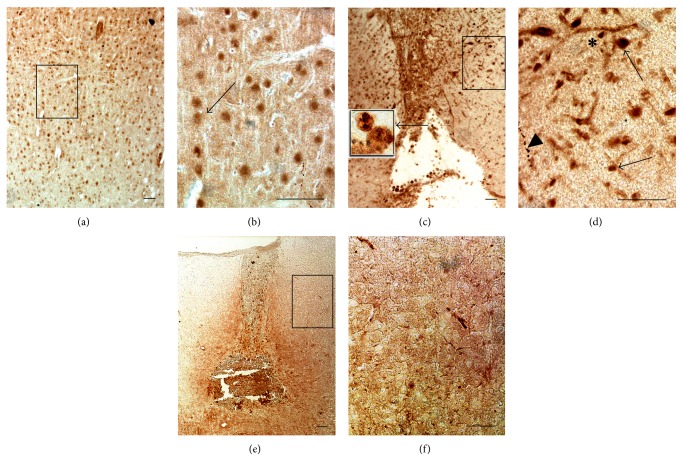
Repetitive HBOT affects CD40 expression after CSI. ((a) and (b)) In control cortical sections CD40 is expressed on neuron-like cells. (c) CSI increased CD40 immunoreactivity in the perilesioned cortex. ((c) inset) High magnification photomicrograph depicts a typical neutrophil with round morphology and multiple nuclei (left up corner of inset), while the enlarged cell in the right corner of inset is probably activated microglia. (d) CD40-positive dark-stained pyknotic neurons, transected axonal processes (denoted with arrows), nerve fiber varicosities (arrow head), and blood vessels (asterisk) were abundantly present in perilesioned area. ((e) and (f)) 10 days of HBOT significantly reduced CD40 immunoreactivity in the injured cortex. Rectangles indicate where the high magnification images are taken from. Scale bar = 50 *μ*m.

Immunohistochemical analysis revealed that CD40 immunoreactivity is constitutively expressed on neuron-like cells. In the control sections, CD40 staining was widely spread throughout the whole cortex, predominantly localized on neuronal cell bodies (Figures 5(a) and 5(b)). After the CSI, strong CD40 immunoreactivity was accumulated within the lesion area (Figure 5(c)). CD40-expressing nonneuronal cells (probably activated microglia and neutrophils) were seen within the necrotic area of the lesion (Figure 5(c), inset). In addition, in the perilesioned cortex most of the CD40 immunoreactivity was found in dark-stained pyknotic neurons and in transected axonal processes (Figure 5(d)). Numerous nerve fiber varicosities with the characteristic appearance of strings of pearls were also stained with anti-CD40 antibody (Figure 5(d), arrow head). In contrast, after 10 days of HBOT a significant reduction of CD40 immunoreactivity was detected within the lesion site and in the injured cortex (Figure 5(e)). Weak immunostaining was associated with a small number of inflammatory infiltrates (microglia and neutrophils) (Figure 5(e)), while staining of neuronal cells was negligible (Figure 5(f)).

**Figure 6:**
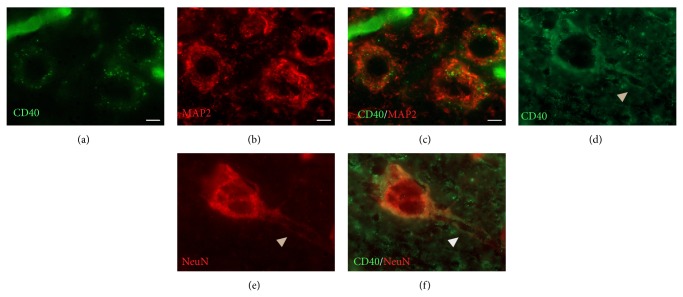
CD-40 (green fluorescence) colocalization with markers of neuronal cells in the injured cortex. ((a)–(c)) Double-immunofluorescence analysis of CD40 (a) and MAP-2 ((b) red fluorescence) revealed that CD40 fluorescence signal was mostly detected on neuronal cell bodies ((c) yellow fluorescence). ((d)–(f)) CD40 (d) and NeuN ((e) red fluorescence) fluorescence colocalized not only on neuronal cell bodies but also on axons (f). Arrow head points to varicosities along axons. Scale bar = 5 *μ*m.

In order to confirm CD40 expression on neuronal cell bodies and dendrites double-immunofluorescence staining for CD40 (green fluorescence, Figure 6(a)) and MAP-2 (red fluorescence, Figure 6(b)) was performed. Obtained data revealed that CD40 fluorescence signal was mostly detected on neuronal cell bodies (Figure 6(c), yellow fluorescence). Double immunofluorescence of CD40 (green fluorescence, Figure 6(d), and NeuN (red fluorescence, Figure 6(e)) showed their colocalization both on neuronal cell bodies and multiple varicosities on axons (Figure 6(f)).

#### 3.3.2. CSI-Induced Overexpression of CD40L Is Downregulated after HBOT

**Figure 7:**
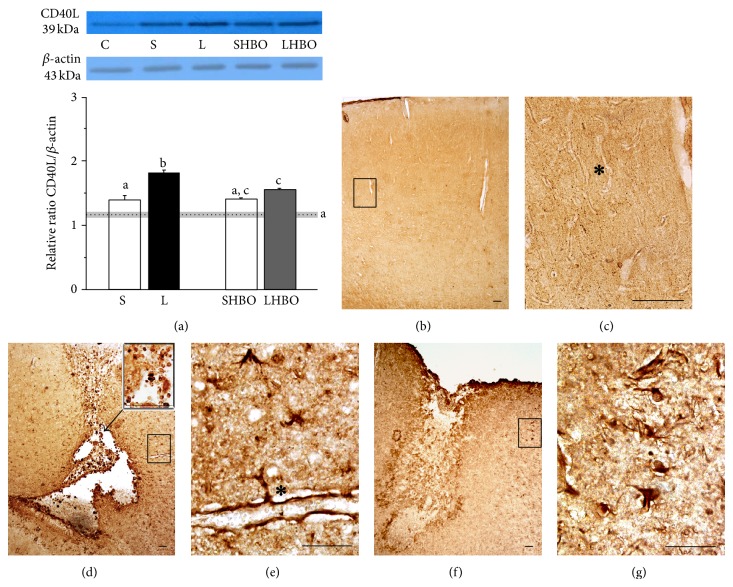
Repetitive HBOT downregulates CD40L expression after CSI. (a) Immunoblot analysis showed that CD40L was present as a single band with a molecular mass of about 36 kDa. After CSI expression of CD40L significantly (*P* < 0.005) increased in respect to intact control (C) and sham-operated animals (S). However, compared to L group in LHBO group statistically significant reduction (*P* < 0.005) of CD40L protein expression was observed. Bars represent mean ± SEM of CD40L protein content (relative to *β*-actin). Samples are from 4 animals per each group. Dot line represents mean of CD40L mRNA or protein level ± SEM (gray area) measured in control animals. Letters indicate significance levels (*P* < 0.005) between lesioned (L) and intact control groups, L versus sham control (S) group, and L compared to lesioned group subjected to the HBO protocol (LHBO). The groups not sharing a common letter are statistically different. Level of significance was analyzed using Student's *t*-test. ((b) and (c)) In control cortex CD40L was localized in blood vessels ((c) asterisk). (d) At 10th day after CSI intense CD40L immunoreactivity occurred in perilesioned area: ((d), inset) in macrophages/microglia and neutrophils and (e) in protoplasmic astrocytes in close contact with endothelial cells via astrocytic end-feet (asterisk). (f) Repetitive HBOT decreased CD40L immunostaining in the injured cortex. (g) CD40L-positive astrocytes had fibrous form. Rectangles indicate where the high magnification images are taken from. Scale bar = 50 *μ*m.

To investigate the effect of HBOT on pattern of CD40L protein expression, left cortices were isolated 10 days after injury. Immunoblot analysis showed that CD40L was present as a single band with a molecular mass of about 36 kDa (Figure 7(a)). There was a significant (*P* < 0.005) increase in CD40L expression following CSI (L group) with respect to intact control (C) and sham-operated animals (S). Compared to L group in LHBO group statistically significant reduction (*P* < 0.005) of CD40L protein expression was observed (Figure 7(a)).

CD40L immunoreactivity was found in blood vessels throughout the whole cortex of control rats (Figures 7(b) and 7(c)). After the CSI, an intense CD40L immunoreactivity occurred in perilesioned area (Figure 7(d)), mostly in hypertrophied astrocytes (Figure 7(e)) and in microglia and neutrophils within the lesion site (Figure 7(d)). Interestingly, some of CD40L-positive hypertrophied astrocytes were in close contact with endothelial cells via astrocytic end-feet (Figure 7(e)). Repetitive HBOT decreased CD40L immunostaining in the injured cortex (Figures 7(f) and 7(g)). CD40L-immunoreactive astrocytes acquired more resting, fibrous form with small cell bodies, and thin, branched processes (Figure 7(g)).

**Figure 8:**
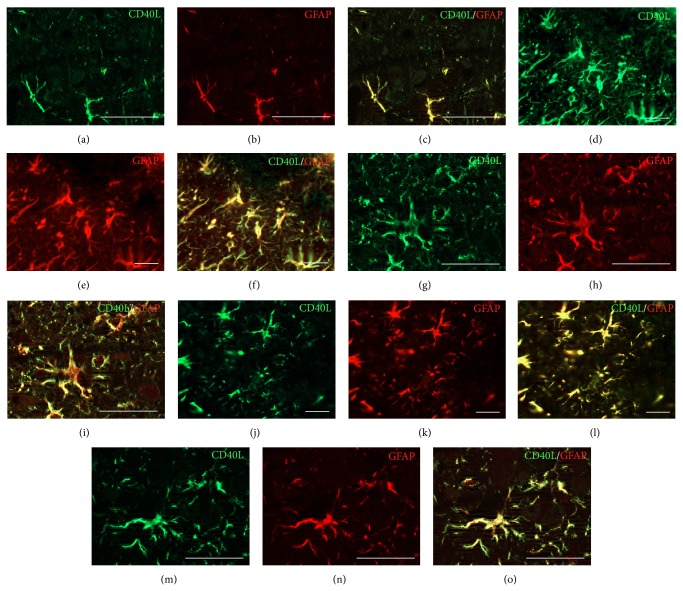
Double-immunofluorescence analysis of CD40L (green) and GFAP (red) colocalization after CSI and HBOT. ((a)–(c)) Sparse CD40L/GFAP positive fibrous astrocytes were seen in the cortex of control rats. ((d)–(f)) Strong CD40L immunoreactivity occurred in GFAP^+^ astrocytes that form dense mesh of glial scar in the area adjacent to the lesion site, providing an overlapping signal (yellow fluorescence). ((g)–(i)) CD40L and GFAP signal is abundantly present at protoplasmic astrocyte cell bodies, and thick proximal and distal processes. ((j)–(l)) Repetitive HBOT decreased intensity of CD40L/GFAP immunofluorescence. ((m)–(o)) Reactive phenotype of astrocytes is transformed into more resting form with smaller cell body and long processes resembling morphology of astrocytes from the control group. Scale bar = 50 *μ*m.

In order to better characterize astrocytic expression of CD40L after CSI and HBOT we performed double-immunofluorescence staining (Figure 8). The CD40L-labeled astrocytes were identified with the antibody against CD40L (green fluorescence) and the astrocytic marker GFAP (red fluorescence). No apparent differences in the pattern of CD40L/GFAP immunostaining between all controls (C, CHBO, S, and SHBO) were noted (data shown as supplemented material). Populations of CD40L/GFAP positive fibrous astrocytes with small cell bodies and thin processes were scattered throughout the cortex of control rats (Figure 8(c)). After the injury, strong CD40L immunoreactivity occurred in GFAP^+^ astrocytes clustered around the lesion site forming dense mesh of glial scar composed of tightly interweaved cell processes. CD40L immunofluorescence completely overlaps with GFAP resulting in yellow fluorescence (Figure 8(f)). This strong CD40L and GFAP immunoreactivity occurred in the hypertrophied cell body, as well as in the thick proximal and distal processes (Figure 8(i)). After repetitive HBOT, reactive phenotype of astrocytes is changed into resting form with smaller cell body and long processes. CD40L is expressed only in subpopulation of fibrous astrocytes around the lesion site (Figures 8(j)–8(l)). The morphology of these astrocytes was similar to astrocytes from control group (Figures 8(m)–8(o)).

## 4. Discussion

Even though HBOT is in use for over the 50 years as a primary or adjunctive therapy, both in experimental and clinical studies, the underlying cellular and molecular mechanisms of hyperbaric oxygenation are still not fully identified [17, 18, 23, 31, 32]. Therefore, in this study we wanted to explore its potential role in suppressing glia-mediated neuroinflammatory response to brain injury. On the basis of obtained data it is evident that repetitive HBOT applied after the cortical injury (1) attenuates reactive astrogliosis and reduces glial scarring, (2) reduces expression of GFAP, vimentin, and ICAM-1 both at gene and tissue levels, and (3) prevents expression of ICAM-1, CD40, and its cognate ligand CD40L on macrophages/microglia, neutrophils, cortical neurons, and reactive astrocytes.

The main concern in HBO therapy is oxygen toxicity that can affect multiple organs, which depends on treatment parameters: pressure and duration of the treatment [17, 33, 34]. In agreement with this knowledge, and according to recommendations of the Committee of the Undersea and Hyperbaric Medical Society [28] and our previous experience [25, 27], we exposed rats to 100% oxygen for 1 h at 2.5 atmospheres absolute for 10 consecutive days. Treatment protocol with 10 exposures to hyperbaric oxygen has been shown to have beneficial effects on improvement in learning and memory in rats with vascular dementia [35]. Using such HBOT protocol, we avoid the potential risk of oxygen toxicity and prevent the appearance of convulsions [36, 37].

### 4.1. Repetitive HBOT Attenuates Reactive Astrogliosis and Glial Scar Formation after CSI

The stab wound injury, applied in this study, creates a discrete and restricted trauma with a necrotic area in the centre of the wound and reactive glial cells bordering the area and is commonly used model for studying the glial response following injury [38]. It is becoming increasingly clear that these reactive glial cells, particularly astrocytes, contribute both to the evolving of tissue damage and to repair of the injured areas [39–41]. After the injury astrocytes become rapidly activated during the process of “reactive astrogliosis” and exhibit phenotypic heterogeneity in molecular expression, morphology, and proliferation, in a manner that is graded with respect to distance from acute focal tissue lesions resulting from traumatic, ischemic, or autoimmune injury [42]. Accordingly, in presented model of cortical stab injury we have demonstrated robust activation and proliferation of astrocytes in the perilesioned cortex. These astrocytes exert pronounced hypertrophy of cell bodies and processes that intertwine and intermingle extensively and form a dense, fibrous glial scar along the boundaries of lesion site. In addition, we have shown that these reactive astrocytes upregulate gene and protein expression of GFAP and vimentin, two intermediate filaments which are prominent cellular hallmarks of reactive gliosis [41]. Repetitive HBOT markedly reduced GFAP and vimentin mRNA expression, almost to the physiological level. At the protein level, HBOT induced statistically significant reduction of GFAP expression, as well. HBOT-induced phenotypic changes of GFAP^+^ and vimentin^+^ astrocytes were maximal adjacent to the damaged tissue, where the majority of reactive astrocytes showed reduced hypertrophy of cell body and had elongated, thin processes that overlap far less extensively or remain within their original territories. Additionally, quantification of luminosity of glial scar composed of GFAP^+^ astrocytes around the lesion site revealed statistically significant reduction of glial scar after HBOT. HBO-induced suppression of microgliosis and astrogliosis was reported to contribute to beneficial effects of HBO treatment in cerebral ischemia [43], neuropathic, and inflammatory pain [44, 45]. Interestingly, Lee et al. [46] reported that prolonged HBOT (for 3 weeks) may increase degree of gliosis indicating that longer oxygen cycling might help in overcoming detrimental effects of gliosis and providing its beneficial effects.

### 4.2. Repetitive HBOT Prevents Spreading of the Neuroinflammation after CSI

The accumulation of neutrophil granulocytes around the site of injury and their infiltration into the injured brain area is crucial for the initiation and progression of inflammation and the extent of secondary brain damage [16, 47]. In our study we have also noted a huge number of neutrophils and activated microglia closely compacted into the lesion core and clustered along the border to the lesion site. ICAM-1 belongs to immunoglobulin superfamily and is critical for the firm arrest and transmigration of leukocytes out of blood vessels and into tissues. Its constitutive expression is documented on endothelial cells and is increased by proinflammatory cytokines [48]. In the present study, we have demonstrated that cortical injury induces upregulation of ICAM-1 mRNA expression. Similar significant and extended induction of ICAM-1 mRNA expression was demonstrated after penetrating ballistic-like brain injury [49] and in the model of focal ischemia [50]. Upregulation of ICAM-1 in cerebral microvessels has been described in several brain pathologies, including brain trauma [50–52]. In our study increased ICAM-1 immunostaining of blood vessels was seen not only in the injured cortex but also on the vessels in contralateral, noninjured cortex pointing to transhemispheric diaschisis, which is usually regarded as functional changes caused by the interruption of input from the lesioned brain region [53]. Our results are in accordance with data obtained by other authors who noticed persistent upregulation of ICAM-1 in contralateral hemisphere as well [50–52, 54]. Further, we have noticed increased ICAM-1 immunoreactivity in a few dark, pyknotic neurons with shrunken neuronal somata, which is consistent with findings of other authors who also found upregulation of ICAM-1 in degenerating neurons [51, 55–57]. One potential explanation for neuronal expression of ICAM-1 is that attachment of leukocytes and/or microglia to healthy neurons could induce neuron injury, either through direct cell-to-cell interaction or by release of potentially cytotoxic substances [51, 56]. More likely, it is suggested that expression of ICAM-1 on degenerating neurons may act as intercellular recognition signal which facilitates their elimination by phagocytotic microglia, neutrophils, natural killer cells, monocytes, and cytotoxic T cells [51, 55, 57]. Therefore, therapeutic interventions aimed on reducing ICAM-1 expression and targeting the passage of immune cells through the BBB via inhibition of cell adhesion molecules present an interesting avenue to dampen the neuroinflammatory response to TBI. Herein, we have demonstrated that HBOT has potential of reducing ICAM-1 expression after CSI both on gene and tissue levels. Thus, injury-induced upregulation of ICAM-1 mRNA expression declined to the control levels following the HBOT. In addition, after HBOT no ICAM-1 immunoreactivity was detected on blood vessels in the injured cortex, while faintly stained ICAM-1 positive neutrophils were spread through lesion core. Several studies have also shown that HBOT reduced the expression of ICAM-1 and adhesion of neutrophils to the endothelium which correlated with improved neurologic outcome [21, 58–61].

CD40/CD40L interaction stimulates the endothelial cells to upregulate the expression of adhesion molecules, including ICAM-1, which then enhances adhesion and the subsequent transmigration of inflammatory cells across the BBB to the sites of inflammation in the CNS, suggesting an important role for the CD40/CD40L dyad in regulating BBB functions [6, 62]. Indeed, CD40 or CD40L deletion has been shown to diminish the ICAM-1 expression on endothelial cells and prevent neurodegeneration [63]. Having all this in mind we further investigate the influence of CSI and HBOT on CD40 and CD40L expression and cellular localization in the injured cortex.

CD40 is a membrane protein that belongs to the tumor necrosis factor (TNF) receptor family and is expressed on a variety of immune and nonimmune cells, suggesting that CD40 plays a much broader role in cellular processes than it was primarily suspected [6, 64]. In control animals we have detected CD40 immunoreactivity throughout the whole cortex, predominantly localized on neuronal cell bodies. Constitutive expression of CD40 by cortical neurons and primary cultured neuronal and neuron-like cells is also reported by Tan et al. [65]. However, after the CSI we have detected strong CD40 immunoreactivity within the lesion area on nonneuronal cells (most likely activated microglia and neutrophils) and vascular endothelium. Our results are consistent with findings which demonstrated remarkable enhancement of CD40 expression by multiple cell types in the CNS, including tissue macrophages, microglia, astrocytes, vascular endothelial, and smooth muscle cells and neurons, indicating a role of CD40 in neuroinflammation [7, 62, 63, 65, 66]. Additionally, we have found that most of the CD40 immunoreactivity in the perilesioned cortex was associated with dark-stained pyknotic neurons and transected axonal processes. Double-immunofluorescence staining with CD40 and MAP-2 revealed CD40 expression on neuronal cell bodies and axons. In addition, as shown by double-immunofluorescence staining for CD40 and NeuN, multiple nerve fiber varicosities were also stained with anti-CD40 antibody suggesting its role in promoting neurodegeneration. Involvement of CD40 in activation of pathogenic cascade that will result in neuronal cell death is documented in a wide variety of neuroinflammatory and neurodegenerative diseases [57, 63, 64, 67]. In the present study we used hyperbaric oxygenation to prevent these CD40-induced pathological processes after brain injury. Indeed, obtained data revealed a significant reduction of CD40 expression in the perilesioned cortex after repetitive HBOT.

CD40 explore its full activation after ligation with its natural ligand, CD40L, which has a broad spectrum of functions [68]. It is suggested that reactive astrocytes are a predominant source of CD40L in the brain [6, 9]. Immunohistochemical data of the current study revealed the presence of very low, constitutive levels of CD40L immunoreactivity in blood vessels, while no CD40L staining occurred in astrocytes in the cortex of intact brain. Stab injury to the brain produced an induction of CD40L protein expression and increased CD40L immunoreactivity in hypertrophied astrocytes throughout the injured cortex, which often were in close association with intensively stained vascular endothelium forming so called “perivascular” end-feet. In addition, CD40L-positive macrophages/microglia and neutrophils were seen within the lesion area. Using double-immunofluorescence CD40L/GFAP staining we detected a strong CD40L immunoreactivity in GFAP^+^ astrocytes clustered around the lesion site which occurred in the hypertrophied cell bodies, as well as in the proximal and distal processes. Our results are consistent with findings of Calingasan et al. [9] who have shown intense CD40L expression in a subset of GFAP-positive astrocytes around the lesion site in the same model of CSI. Considering the ability of CD40L to trigger an inflammatory reaction of vascular endothelial cells [69] the same authors [9] proposed that CD40L from astrocytes may synergize with the enhanced CD40 expression to trigger an inflammatory reaction of endothelial cells. Thus, CD40/CD40L interaction by astrocytes could initiate multiple signaling cascades that lead to the release of key proinflammatory mediators. Importantly, we have found that application of ten successive HBOT markedly reduced CD40L expression in the perilesioned cortex, while CD40L-immunoreactive astrocytes acquired more resting, fibrous form similar to those from control group.

## 5. Conclusion

In summary, this study reveals for the first time that repetitive HBOT attenuates reactive astrogliosis, suppresses formation of glial scar, and prevents activation of CD40/CD40L/ICAM-1 deleterious cascade of events after cortical stab injury. Moreover, HBOT effectively decreased glia-mediated inflammatory response and tissue-damaging effects of neutrophils and prevented neuronal degeneration after CSI. Considering CD40/CD40L and ICAM-1 involvement in amplification of immune response after brain injury, observed reduction of their expression after HBOT confirmed the role of HBOT in attenuation of inflammatory responses after TBI. Thus, HBOT, by limiting production of inflammatory mediators fosters formation of more permissive environment for repair and regeneration.

## Supplementary Material

Supplementary Figure 1: Staining pattern of GFAP, vimentin (VIM) and ICAM-1 in the left cortex of control group (C, intact rats), control HBO group (CHBO, intact rats subjected to the HBO protocol for 10 consecutive days), sham group (S, the animals that underwent surgical procedure without skull opening and sacrificed 10 days post operation) and sham HBO group (SHBO, the animals that underwent sham surgery and were subjected to the HBO protocol for 10 consecutive days). Sham operation and/or the treatment with HBO did not significantly change the GFAP, VIM and ICAM-1 staining pattern. Scale bar = 50 µm.
Supplementary Figure 2: Staining pattern of CD40 and CD40L in the left cortex of control group (C, intact rats), control HBO group (CHBO, intact rats subjected to the HBO protocol for 10 consecutive days), sham group (S, the animals that underwent surgical procedure without skull opening and sacrificed 10 days post operation) and sham HBO group (SHBO, the animals that underwent sham surgery and were subjected to the HBO protocol for 10 consecutive days). Staining pattern of CD40 i CD40L was not significantly changed after the sham operation and/or treatment with HBO. Scale bar = 50 µm.
Supplementary Figure 3: Double immunofluorescence analysis of CD40L (green) and GFAP (red) colocalization in the left cortex of control group (C, intact rats), control HBO group (CHBO, intact rats subjected to the HBO protocol for 10 consecutive days), sham group (S, the animals that underwent surgical procedure without skull opening and sacrificed 10 days post operation) and sham HBO group (SHBO, the animals that underwent sham surgery and were subjected to the HBO protocol for 10 consecutive days). Paucity of CD40L/GFAP (yellow fluorescence) positive fibrous astrocytes was seen throughout the left cortex. Morphology of these astrocytes was not significantly changed after the sham operation and/or treatment with HBO. Scale bar = 50 µm.
