# Supplementary figures and images for: Repetitive Hyperbaric Oxygenation Attenuates Reactive Astrogliosis and Suppresses Expression of Inflammatory Mediators in the Rat Model of Brain Injury
Source: Mediators Inflamm. 2015 Apr 20;2015:498405. doi: 10.1155/2015/498405 (PMC4417949; doi:10.1155/2015/498405)

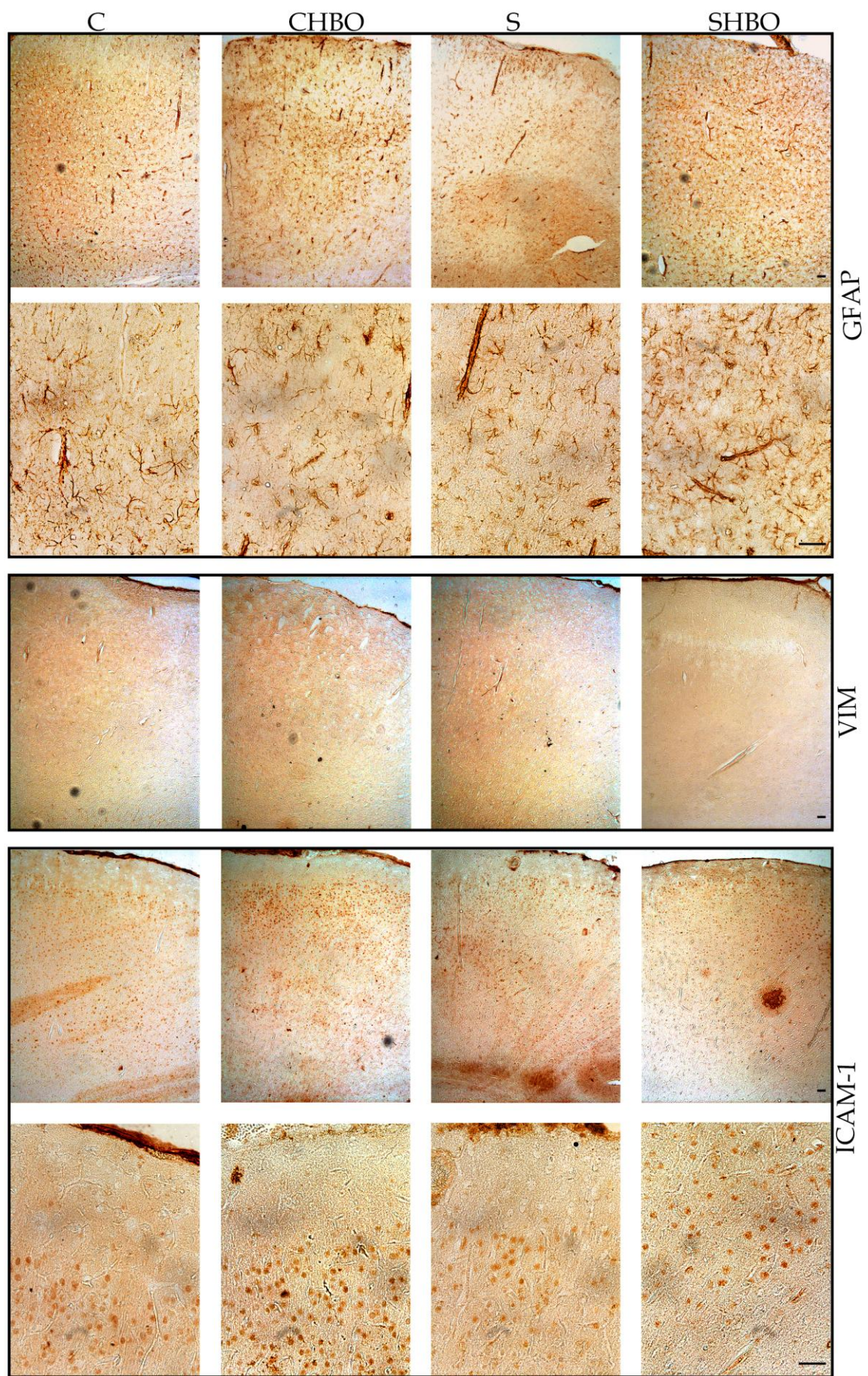

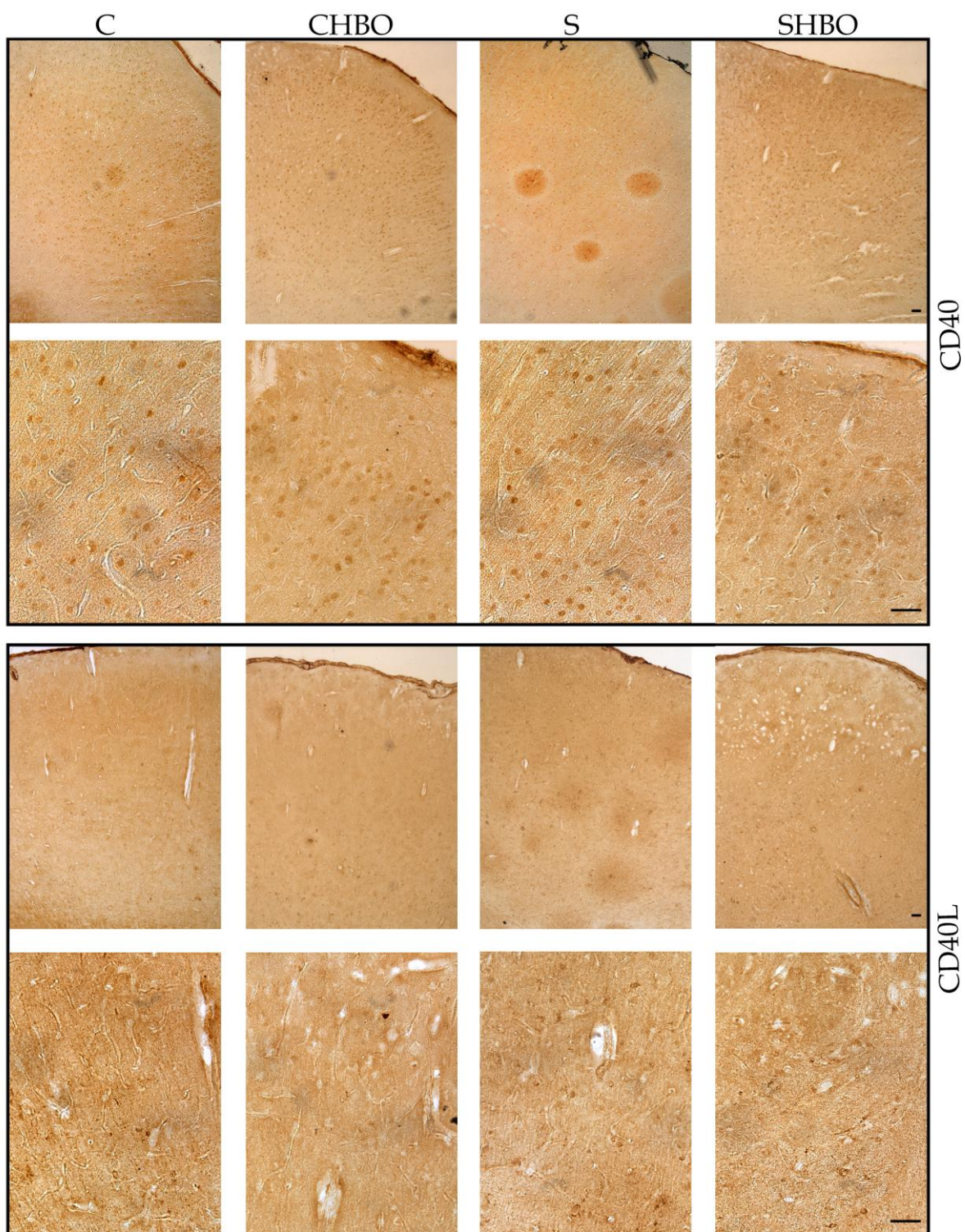

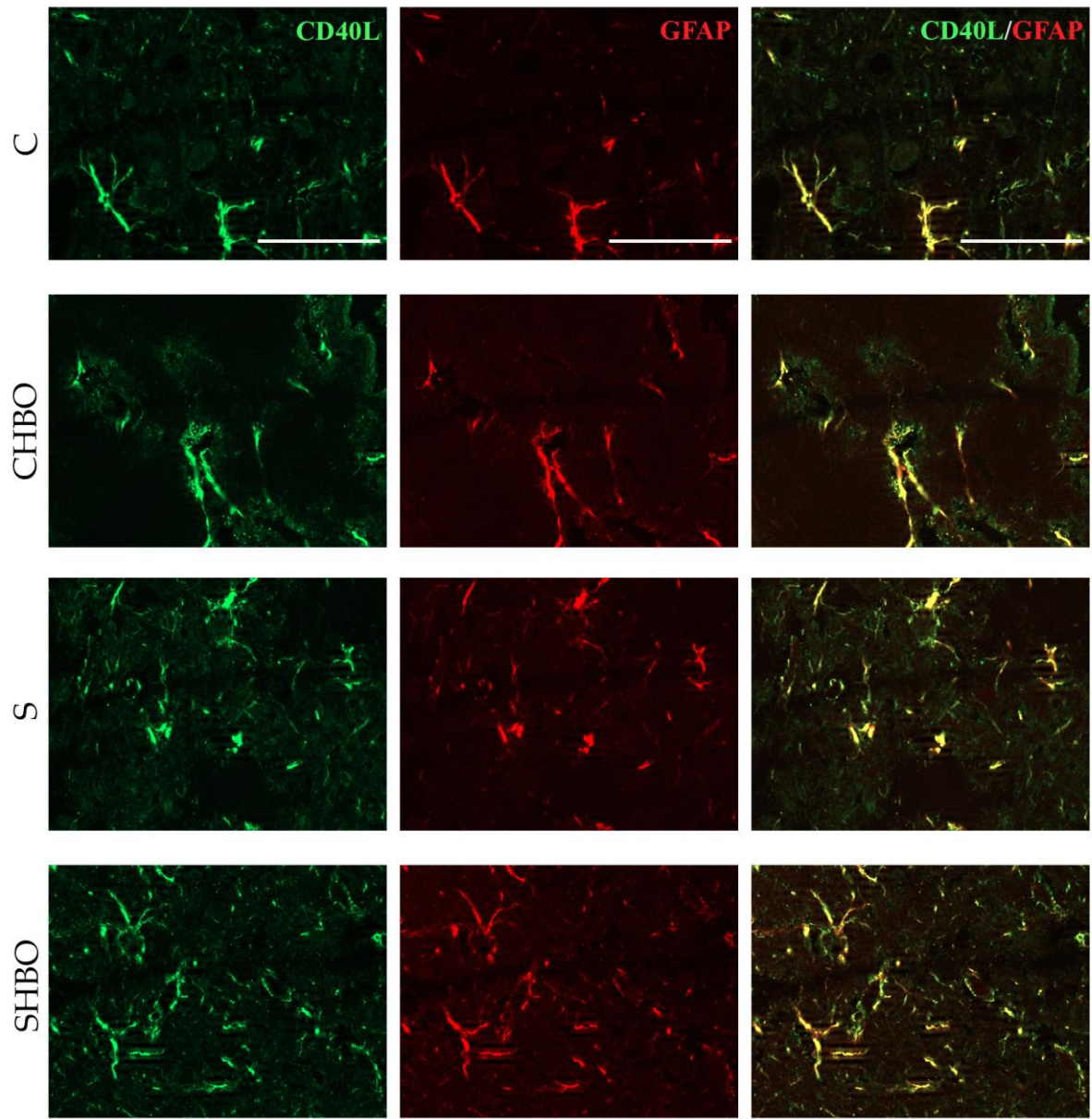

Supplement: Supplementary file 1 — Supplementary Figure 1: Staining pattern of GFAP, vimentin (VIM) and ICAM-1 in the left cortex of control group (C, intact rats), control HBO group (CHBO, intact rats subjected to the HBO protocol for 10 consecutive days), sham group (S, the animals that underwent surgical procedure without skull opening and sacrificed 10 days post operation) and sham HBO group (SHBO, the animals that underwent sham surgery and were subjected to the HBO protocol for 10 consecutive days). Sham operation and/or the treatment with HBO did not significantly change the GFAP, VIM and ICAM-1 staining pattern. Scale bar = 50 µm. Supplementary Figure 2: Staining pattern of CD40 and CD40L in the left cortex of control group (C, intact rats), control HBO group (CHBO, intact rats subjected to the HBO protocol for 10 consecutive days), sham group (S, the animals that underwent surgical procedure without skull opening and sacrificed 10 days post operation) and sham HBO group (SHBO, the animals that underwent sham surgery and were subjected to the HBO protocol for 10 consecutive days). Staining pattern of CD40 i CD40L was not significantly changed after the sham operation and/or treatment with HBO. Scale bar = 50 µm. Supplementary Figure 3: Double immunofluorescence analysis of CD40L (green) and GFAP (red) colocalization in the left cortex of control group (C, intact rats), control HBO group (CHBO, intact rats subjected to the HBO protocol for 10 consecutive days), sham group (S, the animals that underwent surgical procedure without skull opening and sacrificed 10 days post operation) and sham HBO group (SHBO, the animals that underwent sham surgery and were subjected to the HBO protocol for 10 consecutive days). Paucity of CD40L/GFAP (yellow fluorescence) positive fibrous astrocytes was seen throughout the left cortex. Morphology of these astrocytes was not significantly changed after the sham operation and/or treatment with HBO. Scale bar = 50 µm. [file 498405.f1.pdf]
